# Cardiac metastasis from squamous cell carcinoma of the oral cavity: A rare case report

**DOI:** 10.1016/j.radcr.2024.10.139

**Published:** 2024-11-25

**Authors:** Mark Marreros, Nicolas Dulguerov, Maxime Mermod

**Affiliations:** a Maxillo-Facial Surgery Unit, Geneva University Hospital, Gabrielle-Perret-Gentil 4, 1205 Genève, Switzerland; b Head and Neck Surgery Unit, ENT Department of Geneva University Hospital, Gabrielle-Perret-Gentil 4, 1205 Genève, Switzerland

**Keywords:** Cardiac metastasis, Head and neck, Squamous cell carcinoma, Immunotherapy, Oncology

## Abstract

A 58-year-old male with squamous cell carcinoma of the floor of mouth underwent surgical planning for tumor resection and floor of mouth reconstruction. Unexpectedly, preoperative cervico-thoracic computed tomography (CT) indicated possible right ventricular intramural thrombosis, prompting initiation of unfractionated heparin. Follow-up echocardiography revealed no thrombus reduction, raising concerns of intracardiac metastasis. Positron emission tomography with 2-deoxy-2-[fluorine-18]fluoro- D-glucose with computed tomography (^18^F-FDG PET/CT) showed a hypermetabolic mass in the right ventricle, raising concern for a distant tumor metastasis. Under anti-coagulation, the patient experienced recurrent tumor-related hemorrhage, necessitating urgent lingual artery embolization. Due to disease progression, surgical options were dismissed in favor of palliative chemo-immunotherapy, which both led to significant regression of both primary and metastatic lesions.

## Introduction

Intracardiac metastases are rare, with an incidence of 2.3% to 18.3% reported in autopsy studies [[1], [2], [3]]. Primary tumors commonly originate from breast cancer, melanoma, leukemia, malignant germ cell tumors, and malignant thymoma [2]. Metastasis from head and neck malignancies to the heart is particularly rare, reflecting the limited literature available. Herein, we report a case of unexpected cardiac metastasis in a patient with advanced squamous cell carcinoma of the oral cavity.

## Case presentation

**Fig. 1:**
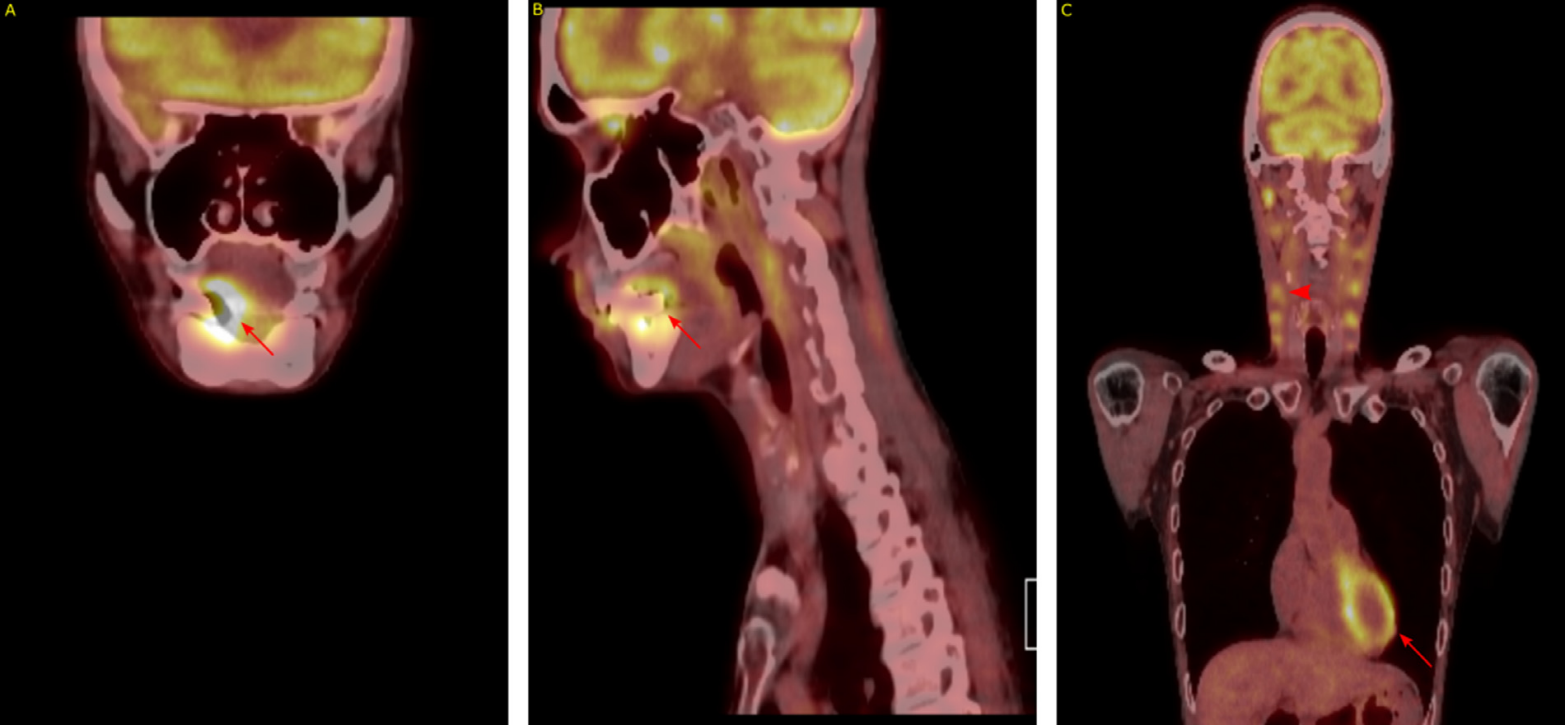
Panel A: Frontal view of primary lesion involving the right side of the tongue (red arrow). Panel B: Sagital view of primary lesion involving the right side of the tongue (red arrow). Panel C: Frontal view showing bilateral adenopathy (red arrowhead) and physiological cardiac uptake with no visible metastasis (red arrow).

A 58-year-old smoker experienced sublingual discomfort and swelling, which went unaddressed due patient's concerns for a worrisome diagnosis. The patient finally sought emergency Otolaryngology consultation due to onset of aphagia, odynophagia, bloody sputum, a 12 kg weight loss, and asthenia over the previous 3 weeks. Physical examination revealed a 4 cm ulcerative lesion on the right tongue and adjacent gum, with right-sided firm, level Ib lymphadenopathy. Biopsy identified a p16-negative squamous cell carcinoma with high PD-L1 expression (CPS > 50). ^18^F-FDG PET/CT imaging in July 2023 showed a hypermetabolic mass on the right side of the tongue with invasion into the right submandibular gland and focal bone invasion in the para-symphyseal region of the mandible, accompanied by bilateral necrotic adenopathy impinging on the right jugular vein (Fig. 1).

Tumor was staged as cT4aN2cM0. The curative strategy comprised a major composite oral cavity resection and modified radical bilateral neck dissection. Surgical reconstruction was planned for a double free flap with a fibula free flap for the bone component, and an antero-lateral free flap for the soft tissue element of the reconstruction.

**Fig. 2:**
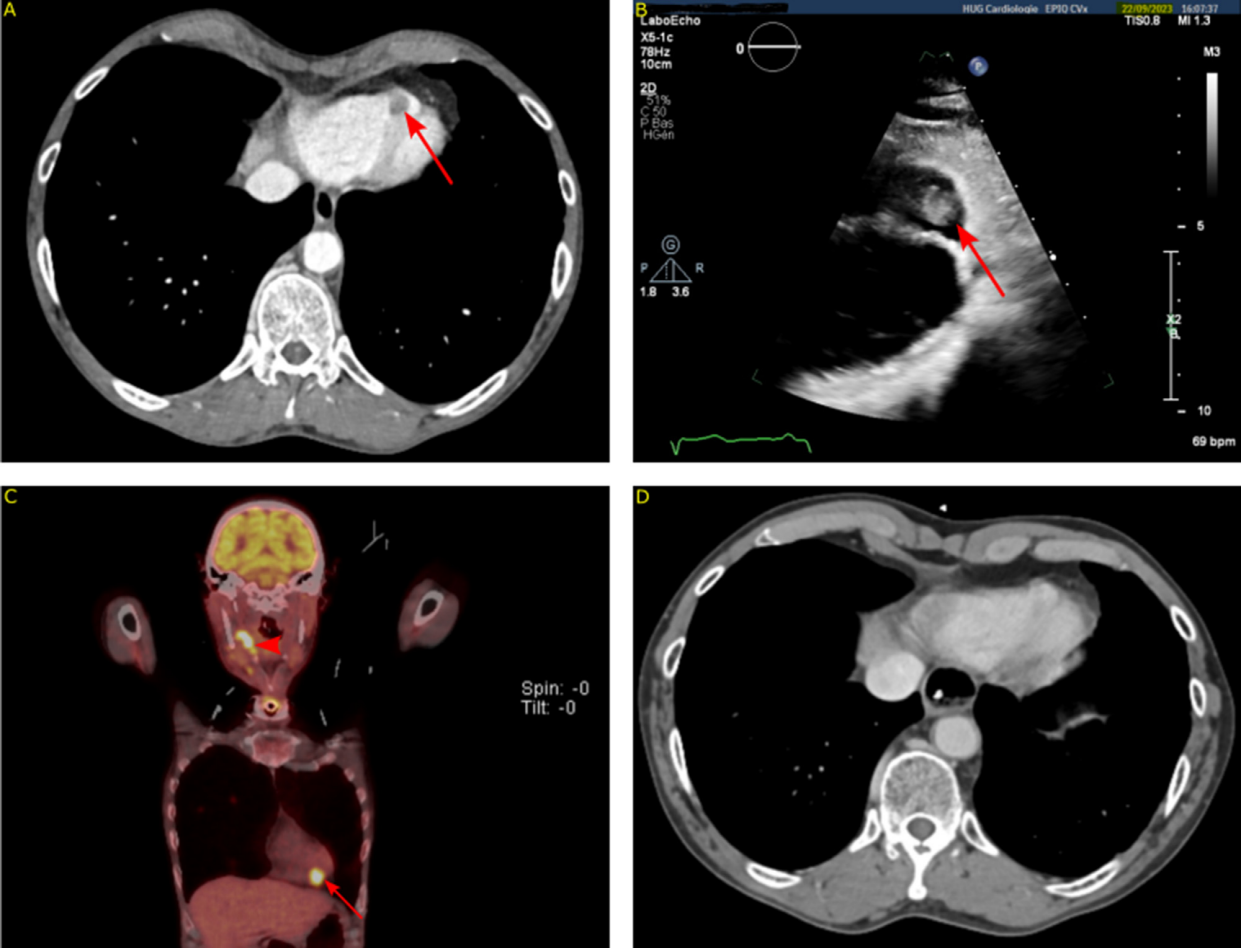
Panel A: Initial CT-scan showing an intraventricular mass (red arrow). Panel B: Echocardiography showing right ventricular lesion (red arrow). Panel C: Coronal view of initial ^18^F-FDG PET/CT showing primary cancer hypermetabolism (arrowhead) and cardiac metastasis (arrow). Panel D: Follow-up CT showing a marked regression in size of intracardiac lesion at 6 months after treatment initiation.

The patient was admitted for nutrition and management of intermittent tumoral bleeding. A presurgical thoracic and neck CT scan revealed a right ventricular mass, suspected to be a thrombus (Fig. 2, Panel A). This prompted anticoagulation with unfractionated heparin (UFH), following echocardiography (Fig. 2, Panel B) and cardiological consultation. However, heparin exacerbated tumoral bleeding, leading to hemorrhagic shock on September 27, 2023, managed by embolizing the right lingual artery. Consequently, surgery was postponed. Subsequent echocardiography indicated no thrombus resolution and persistent right ventricular hypokinesia. Cardiological review raised suspicions of metastasis due to the mass's appearance and unresponsiveness to anticoagulation, leading to discontinuation of heparin. A ^18^F-FDG PET/CT was performed to evaluate the cardiac lesions, revealing intense hypermetabolic activity of the right ventricular mass, consistent with metastasis. Additionally, significant progression of the primary lesion and multiple bilateral necrotic lymph nodes were observed (Fig. 2, Panel C). After this, a protective tracheostomy was performed on September 29, 2023, in anticipation of potential airway obstruction from immunotherapy-related edema. The oncological multidisciplinary board reassessed the patient's status postembolization and, given the concern for distant metastasis, deemed surgery unreasonable. The decision was made to proceed with palliative chemo-immunotherapy, combining carboplatin and pembrolizumab. A follow-up thoraco-abdominal scan 6 months later indicated a reduction in the size of the right ventricular mass and primary lesion (Fig. 2, Panel D).

## Discussion

Secondary cardiac involvement is uncommon, much like primary cardiac tumors. K Y Lam et al. reviewed 12,485 autopsies over 20 years at Queen Mary Hospital in Hong Kong, finding a 1.23% incidence of secondary tumors [4]. The most frequent primary sources were lung (31.7%) and esophageal carcinoma (28.7%) in males, and lung (35.9%) and lymphoma (17.0%) in females. Similarly, K. P. Abraham et al. found an 11.8% incidence of cardiac tumor involvement in 3,314 autopsies, predominantly from lung cancer and lymphoma [5].

A literature search on PubMed for head and neck tumors with cardiac metastases yielded 4 case reports (Table 1). Suha Abu Khalaf et al. reported a Stage III tonsil SCC with cardiac involvement presenting initially as stroke and limb ischemia from peripheral embolization [6]. Histopathological examination during limb thrombectomy confirmed the presence of tumor cells, with definitive diagnosis by autopsy. Huang Chung Chen et al. described a stage IVb SCC of the right lower gum presenting with tamponade; diagnosis was supported by elevated carcinoembryonic antigen levels during pericardiocentesis, despite negative cytology [7].

**Table 1:** This table summarize the articles found in our review of the current literature regarding cardiac metastasis of head and neck primary malignancies.

Article	Author	Sex	Age	Primary tumor	Secondary	TNM	Stage	Clinical presentation	Diagnostic	Intervention	Treatment
Intracardiac metastasis from head and neck squamous cell carcinoma	Suha Abu Khalaf	M	57	Right tonsil SCC		pT1pN2bM0	III	ischemic stroke, peripheric ischemia	US, autopsy	Transoral robotic right tonsillectomy + neck dissection	Cisplatin + RTH + Immunotherapy
Unusual sites of metastatic involvement: intracardiac metastasis from laryngeal carcinoma	Alhakeem and al.	M	49	Laryngeal cell carcinoma	VG, VD			Peripheric embolization (RLL)	TTE, CT scan, immunostaining peripheric embolization	Resection	RTH
Intracardiac metastasis of high-grade sarcoma of the neck causing right ventricular outflow obstruction	B. Thyagarajan and al.	M	71	high-grade sarcoma of the neck	Pulmonaire, VD obstructif			Dyspnea	TTE, CT scan, MRI		nihil
Biventricular ‘salmon roe’ lesions: intracardiac metastasis from oral squamous cell carcinoma	Huang Chung Chen and al.	F	43	RLQ gingival SCC	VD, VG, lung, liver, hilar lymph node		IVb	Orthopnea, pericardial effusion	Pericardiocentesis (CAE)		

SCC, Squamous cell carcinoma; RLQ, Right lower quadrant; LV, left ventricle; RV, right ventricle; RLL, right lower limb; US, Ultrasound; TTE, Transthoracic echocardiography, CEA, Carcinoembryonic antigen, RTH, Radiotherapy.

Thyagarajan et al. detailed a case involving a 71-year-old man with an undifferentiated soft tissue sarcoma of the neck and lung metastases, presenting with exertional dyspnea. Imaging revealed a right ventricular mass partially obstructing the outflow tract, identified by CT and echocardiography [8].

Intracardiac metastasis poses diagnostic challenges due to its rarity and clinical mimicry of other cardiovascular conditions. In our case, significant morbidity resulted from bleeding caused by anticoagulation of a lesion misidentified as a mural thrombus. Diagnosis was eventually confirmed via ^18^F-FDG PET/CT, a critical yet resource-intensive tool for both diagnosis and monitoring.

## Conclusion

Secondary cardiac involvement from head and neck tumors is rare, with limited available literature. In cases where a cardiac mass suspected to be thrombotic does not respond to anticoagulation, ^18^F-FDG PET/CT is a valuable diagnostic tool to assess for potential malignancy. Furthermore, ^18^F-FDG PET/CT is also beneficial for monitoring therapeutic responses, making it an essential imaging technique in the management of such complex cases.

## Patient consent

I hereby confirm that a written informed consent for publication was obtained from the patient whose case we describe in this manuscript.
